# Early physiological indicators of narcissism and self‐esteem in children

**DOI:** 10.1111/psyp.14082

**Published:** 2022-05-03

**Authors:** Eddie Brummelman, Milica Nikolić, Barbara Nevicka, Susan M. Bögels

**Affiliations:** ^1^ Research Institute of Child Development and Education University of Amsterdam Amsterdam the Netherlands

**Keywords:** childhood, heart rate, heart rate variability, narcissism, self‐esteem, skin conductance

## Abstract

A common belief is that narcissism is a manifestation of high self‐esteem. Here, we argue that self‐esteem and narcissism are fundamentally distinct and have unique early physiological indicators. We hypothesized that children predisposed to narcissism would show *elevated*, whereas children predisposed to high self‐esteem would show *lowered*, physiological arousal in social‐evaluative contexts. We tested this in a prospective study including 113 children, who were first assessed at age 4.5, a critical age when children begin evaluating themselves through others' eyes. At age 4.5, children sang a song in front of an audience while being videotaped. Children's physiological arousal (skin conductance, heart rate, and heart rate variability) was assessed while children anticipated, performed, and recovered from the singing task. At age 7.5, children's narcissism and self‐esteem levels were assessed. Consistent with our predictions, children predisposed to higher narcissism levels showed elevated skin conductance levels during anticipation. Their skin conductance levels further rose during performance (but less so than for other children) and failed to return to baseline during recovery. By contrast, children predisposed to higher self‐esteem levels showed lowered skin conductance levels throughout the procedure. The effects emerged for skin conductance but not heart rate or heart rate variability, suggesting that arousal was sympathetically driven. Effects were larger and more robust for self‐esteem than for narcissism. Together, these findings uncover distinct physiological indicators of narcissism and self‐esteem: Narcissism is predicted by indicators reflecting early social‐evaluative concerns, whereas self‐esteem is predicted by indicators reflecting an early sense of comfort in social‐evaluative contexts.

## Abstract 2

Some experts fear that self‐esteem can develop into narcissism. Challenging this view, we show that self‐esteem and narcissism (at age 7.5) have distinct early physiological indicators (at age 4.5). In our prospective study, narcissism was predicted by elevated, whereas self‐esteem was predicted by lowered, physiological arousal in a social‐evaluative context.

## 1 INTRODUCTION

Since the 1960s, with the rise of individualism, society has become increasingly focused on children's self‐esteem (Brummelman & Sedikides, 2020). This is understandable given the benefits of self‐esteem for children's adjustment (Orth & Robins, 2014). A growing body of literature shows that children with higher self‐esteem, on average, experience less anxiety and depression (Sowislo & Orth, 2013), behave less aggressively (Donnellan et al., 2005), and perform better in school (Zheng et al., 2020). Yet, some researchers believe that elevated levels of self‐esteem can resemble narcissism: a sense of superiority and entitlement (Baumeister et al., 2003). Challenging this view, we argue that self‐esteem and narcissism are fundamentally distinct, and that each has unique early physiological indicators. In this prospective study, we tested the hypothesis that narcissism and self‐esteem are predicted by distinct early‐childhood patterns of physiological arousal during social exposure.

### 1.1 Separating self‐esteem from narcissism

Self‐esteem is defined as a sense of one's worth as a person (Orth & Robins, 2014), whereas narcissism is defined as a sense of superiority and entitlement (Krizan & Herlache, 2018). In its extreme form, narcissism can manifest as a narcissistic personality disorder, which is rarely diagnosed in minors (American Psychiatric Association, 2013). However, we study narcissism as a non‐clinical, everyday personality trait that is normally distributed in the general population (Thomaes & Brummelman, 2016). We focus on grandiose (rather than vulnerable) narcissism, which is characterized by boldness, extraversion, and boastfulness (Derry et al., 2020; Miller et al., 2017). A common but misguided belief is that narcissism is an extreme form of self‐esteem. In the early days of psychology, psychologists often used the terms narcissism and self‐esteem interchangeably (Pulver, 1986). Since then, psychologists have often characterized narcissism as an inflated, exaggerated, or excessive form of self‐esteem: “the dark side of high self‐esteem” (Baumeister et al., 1996, p. 5). These labels suggest that self‐esteem represents a continuum, with narcissism at its upper end. If these views are correct, then narcissism and self‐esteem should correlate highly and there should be no individuals who have high narcissism but low self‐esteem levels. Contrary to these predictions, narcissism and self‐esteem are only modestly correlated (Campbell et al., 2002; Thomaes et al., 2008), and this correlation becomes even weaker when researchers use more valid measures of narcissism and self‐esteem (Brown & Zeigler‐Hill, 2004) and when they encourage individuals with high narcissism levels to report their self‐esteem truthfully (Myers & Zeigler‐Hill, 2012). In fact, when looking at individuals with high narcissism levels, there are about as many who have high self‐esteem as those who have low self‐esteem (Brummelman et al., 2016; Nelemans et al., 2017). Thus, it is perfectly possible for children with high levels of narcissism to have low levels of self‐esteem. When individuals with high narcissism levels do report high self‐esteem, their self‐esteem tends to be fragile and unstable (Geukes et al., 2017; Rhodewalt et al., 1998; Zeigler‐Hill et al., 2010). Collectively, these findings suggest that self‐esteem and narcissism are distinct.

Stable individual differences in self‐esteem and narcissism tend to emerge around the age of 7 (Thomaes & Brummelman, 2016). At this age, children have acquired two critical cognitive abilities. First, children this age can form global evaluations of their worth as a person (e.g., “I like myself”; Harter, 2012). Second, children this age can use social comparisons for the purpose of self‐evaluation (e.g., “I am better than others”; Gürel et al., 2020; Ruble & Frey, 1991). Although both abilities may emerge earlier (e.g., in the preschool years; Cimpian, 2017; Cimpian et al., 2017), there is yet no substantial evidence that these abilities generate stable individual differences in self‐esteem and narcissism at this younger age. Thus, from the age of 7, individual differences in self‐esteem and narcissism can be assessed reliably (Harter, 2012; Thomaes et al., 2008). Once self‐esteem and narcissism have emerged, they tend to remain relatively stable over time (De Clercq et al., 2017; Trzesniewski et al., 2003).

### 1.2 Early indicators of self‐esteem and narcissism

Despite emerging around the age of 7, it is possible that self‐esteem and narcissism have certain indicators that surface earlier in development, before the traits themselves emerge. It is generally assumed that stable individual differences, including self‐esteem and narcissism, are rooted in temperamental traits that have a biological basis, emerge early in life, and remain relatively stable over time (Shiner, 2005). Clinical case studies and observational research suggest that, even before self‐esteem and narcissism have fully developed, children can show behaviors that can forecast their later self‐esteem and narcissism levels (e.g., Bleiberg, 1984; Harter, 1990; Kernberg, 1989). For example, in one prospective study, children's temperamental traits at age 3 and 4—such as their desire to be at the center of attention and their tendency to overreact to minor frustrations—predicted narcissism levels at ages 14, 18, and 23 (Carlson & Gjerde, 2009). In another prospective study, similar temperamental traits at ages 1 to 3 did not predict self‐esteem levels at ages 40 and 50 (Blatný et al., 2015). Thus, narcissism and self‐esteem seem to have unique early indicators.

What, then, separates the indicators of self‐esteem from those of narcissism? To address this question, we build on social‐cognitive developmental theories of self‐esteem and narcissism (e.g., Brummelman & Sedikides, 2020; Tracy et al., 2009). These theories hold that self‐esteem and narcissism are rooted in distinct sets of socially relevant mental representations (e.g., beliefs, emotions, action tendencies). Broadly, these theories characterize self‐esteem as a secure sense of worth, and narcissism as a fragile sense of superiority. These individual differences arise early in development and manifest physiologically in social‐evaluative contexts. They manifest physiologically because the human nervous system is programmed to handle threats, not only physical threats, but also social threats, such as negative evaluation (e.g., Dickerson, 2008; MacDonald & Leary, 2005). Indeed, social‐evaluative contexts share a common element: They pose a threat to the fundamental goal of maintaining a positive self in the eyes of others (e.g., Bowlby, 1969; Leary & Baumeister, 2000; Rochat, 2009).

Specifically, we theorize that self‐esteem is characterized by reduced, whereas narcissism is characterized by increased, social‐evaluative concerns. Children with high self‐esteem levels are generally satisfied with themselves (Harter, 2012). They see themselves as intrinsically worthy and tend to assume that others value them for who they are (Leary & Baumeister, 2000), even when they fail to live up to others' expectations of them (Baldwin & Sinclair, 1996; Brummelman & Sedikides, 2020). As such, children with high self‐esteem levels tend to believe that others value them unconditionally (Kernis et al., 2000), and they tend to be securely attached to others (Menon et al., 2018). Consequently, they tend to not fear other people's evaluations of them (Thomaes et al., 2008).

By contrast, children with high narcissism levels feel superior to others, but they do not necessarily see themselves as intrinsically worthy. Unlike children with high self‐esteem levels, children with high narcissism levels assume that others value them only when they live up to others' expectations of them (Tracy et al., 2009). Children with high narcissism levels tend to believe that others value them conditionally (Assor & Tal, 2012; Curran et al., 2017), and they tend to be insecurely attached (Menon et al., 2018). Unsurprisingly, these children often employ strategies to elicit positive social evaluations (Grapsas et al., 2020; Morf & Rhodewalt, 2001). For example, they try to be at the center of attention, brag about themselves, and show‐off (Thomaes & Brummelman, 2016). When children with high narcissism levels do not receive the positive evaluations they desire, they may feel disappointed in themselves (Thomaes et al., 2010) and even blush—a hallmark of shame (Brummelman et al., 2018). Thus, unlike children with high self‐esteem levels, children with high narcissism levels tend to be worried about the impressions they make on others. Supporting this view, children with high narcissism levels report an elevated fear of negative evaluation (Thomaes et al., 2008), which is captured by items such as “I worry about what other children say about me” (Greca & Stone, 1993).

We theorize that these individual differences in social‐evaluative concerns can arise early in development. By their second birthday, children are already sensitive to social evaluation (Botto & Rochat, 2019). Over the next few years, children learn to act strategically to elicit positive social evaluations (Heyman et al., 2021; Silver & Shaw, 2018). From the age of 4, children are aware that others might evaluate them positively or negatively (Burhans & Dweck, 1995). They start evaluating themselves through the eyes of others, estimating whether they are evaluated positively or negatively (Selman, 1980). At the same time, children start to realize that others' evaluations of them can be conditional on their behaviors and achievements (Burhans & Dweck, 1995). As a result, they can experience intense social‐evaluative concerns, even in the absence of explicit evaluations by others (Lewis, 2003; Nikolić et al., 2016). By assessing children's social‐evaluative concerns at this critical age, we may be able to identify early indicators of later self‐esteem and narcissism in children.

### 1.3 Capturing social‐evaluative concerns

Together, these lines of research are consistent with our hypotheses derived from social‐cognitive developmental theories, namely that self‐esteem is characterized by reduced, whereas narcissism is characterized by increased, social‐evaluative concerns. However, capturing social‐evaluative concerns in young children can be challenging, because not all young children are able to verbalize these concerns (Luby et al., 2007) and children with high narcissism levels may deny or suppress social‐evaluative concerns, especially if those concerns would reveal something fragile or vulnerable about them (Brummelman et al., 2018). We therefore used physiological measures of children's arousal in a social‐evaluative context. Such measures do not rely on children's verbal abilities and circumvent narcissistic impression management strategies.

Consistent with our hypotheses, evidence links self‐esteem to reduced physiological arousal in social‐evaluative settings. For example, when adults with high self‐esteem levels are socially rejected, they show reduced cortisol reactivity (Ford & Collins, 2010), which suggests reduced stress levels (Miller et al., 2007). Similarly, when adults with high self‐esteem levels perform a challenging task in front of a critical audience, they show reduced cortisol reactivity (Pruessner et al., 1999; Pruessner et al., 2005) as well as reduced activity in the neural stress system (Kogler et al., 2017). These findings suggest that individuals with high self‐esteem levels experience reduced social‐evaluative concerns, which can be captured physiologically.

By contrast, evidence links narcissism to elevated physiological arousal in social‐evaluative settings. Although there is yet no consistent evidence that narcissism is related to chronically elevated stress hormone levels (Wardecker et al., 2018), there is some evidence to suggest that individuals with high narcissism levels show elevated stress in social‐evaluative contexts. For example, when adults with high narcissism levels give a presentation in front of an audience, they tend to show elevated cortisol levels (Edelstein et al., 2010); and when they experience emotional distress, they tend to show elevated alpha‐amylase levels (Cheng et al., 2013). Both responses reflect acute psychosocial stress (Miller et al., 2007; Rohleder et al., 2004). Similarly, when adults with high narcissism levels are ostracized by others, they tend to show increased activity in the social‐pain areas of the brain (Cascio et al., 2015). Also, when children with high narcissism levels lose status among peers, they tend to show increased corrugator supercilii activity (i.e., frowning), which reflects negative affect (Grapsas et al., 2021; also see Grapsas et al., 2022). Although pertaining to different age groups and distinct biological systems, these findings converge to suggest that individuals with high narcissism levels experience elevated social‐evaluative concerns, which can be captured physiologically.

Building on and extending these findings, our aim was to capture young children's social‐evaluative concerns by assessing their physiological arousal in a prototypical social‐evaluative context: performing on stage in front of an audience while being videotaped (Dickerson, 2008). This task is known to elicit arousal in children (Seddon et al., 2020) but more so in some children than in others, which enables us to investigate individual differences in children's responses to this prototypical social‐evaluative context (Krämer et al., 2012; Schmitz et al., 2011; Tuschen‐Caffier et al., 2011). We measured children's skin conductance, heart rate, and heart rate variability, because these processes are implicated in stress and anxiety (Appelhans & Luecken, 2006; Chen & Drummond, 2008). When children are impacted by stress or anxiety, they tend to experience increased skin conductance (i.e., sweating), increased heart rate, and reduced heart rate variability. Reduced heart rate variability is thought to reflect dysregulated emotional responding (Appelhans & Luecken, 2006). Each process reflects a distinct biological mechanism, with skin conductance reflecting sympathetic activation, heart rate variability reflecting parasympathetic withdrawal, and heart rate reflecting a mix of sympathetic activation and parasympathetic withdrawal (Kreibig, 2010). Unsurprisingly, then, the associations between these physiological measures tend to be weak (Dieleman et al., 2015; Fabes et al., 1993), which shows that they capture different aspects of the same stress response. Thus, by assessing these processes simultaneously, we provide a comprehensive understanding of children's physiological arousal in a social‐evaluative context.

Moving beyond prior research in adults, our aim was *not* to identify cross‐sectional physiological correlates of narcissism and self‐esteem. Rather, our aim was to identify early physiological indicators of narcissism and self‐esteem (i.e., physiological indicators that can be detected at an age before narcissism and self‐esteem emerge). This has never been done before, so our study is the first to identify physiological precursors (rather than merely correlates) of narcissism and self‐esteem. We used a prospective research design, so that we could examine whether and how physiological arousal in a social‐evaluative context in early childhood (age 4.5) would predict individual differences in narcissism and self‐esteem 3 years later (age 7.5), the age at which such individual differences first emerge (Thomaes & Brummelman, 2016).

### 1.4 Present study

Using a prospective design, we examined, for the first time, the early physiological indicators of self‐esteem and narcissism in children. At age 4.5, children stood on a stage and sang a song. We assessed children's skin conductance, heart rate variability, and heart rate while children anticipated, performed, and recovered from the singing task. These physiological measures have high temporal precision, allowing us to examine how arousal levels change from before to after the singing task. Three years later, at age 7.5, we assessed children's narcissism and self‐esteem levels.

We hypothesized that, in a prototypical social‐evaluative context, children predisposed to high narcissism levels would show *elevated* physiological arousal, whereas children predisposed to high self‐esteem levels would show *lowered* physiological arousal. We had no a priori hypotheses about whether these associations would be similar or different across physiological measures (i.e., skin conductance, heart rate variability, and heart rate) and task phases (i.e., anticipation, performance, and recovery). We did explore whether associations would differ between physiological measures and task phases, because this offers a completer and more nuanced picture of early physiological indicators of narcissism and self‐esteem.

## 2 METHOD

### 2.1 Participants

Participants were 113 children (87% Dutch origin, 53% girls) who took part in a larger longitudinal study (for a detailed description of the sample and measures, see de Vente et al., 2011), including all children who completed the 4.5 or the 7.5 years measurement. Families were recruited during the pregnancy with a first child through midwives, advertisements in magazines, and leaflets at pregnancy courses and baby shops. All parents spoke Dutch or English fluently. Children could not participate if their birth weight was < 2000 g or if they had any neurological deficits. Parents (93% of Dutch origin, ages 24–64; *M* = 37.14, *SD* = 4.36) had a relatively high educational level (*M* = 6.84, *SD* = 1.16, range: 1 = *primary education*, 8 = *university*). The study was approved by the Ethics Review Board of the University of Amsterdam. Parents provided active informed consent for themselves and their child. Our study was not preregistered. We report all data exclusions (if any) and all measures we analyzed to answer the current research question.

An a priori power analysis, conducted with the SimR package in R for multilevel models, using Monte Carlo simulation (Arend & Schäfer, 2019; Green & MacLeod, 2016), with a small‐to‐medium expected effect size (*r* = .21; see meta‐analysis by Richard et al., 2003) and three repeated measures within individuals (i.e., anticipation, performance, and recovery) showed that we needed 105 participants to obtain a power of (1 – β) = .80 at α = .05, two‐tailed. Despite our directional hypotheses, we used two‐tailed testing to provide a conservative test of our hypotheses.

Due to attrition, each of our main analyses was conducted with a total of 71 children (see 2.3 Data Analysis). Of these children, 88% were of Dutch origin and 54% self‐identified as a girl, and their parents (93% of Dutch origin; ages 26–64; *M* = 37.14, *SD* = 4.52) had a relatively high educational level (*M* = 6.82, *SD* = 1.14, range: 1 = *primary education*, 8 = *university*).^1^ There was no significant difference between the full sample and this final sample in terms of children's sex, *χ* ^2^ (1, *N* = 113) = 0.08, *p* = .779, or country of origin, *χ* ^2^ (1, *N* = 113) = 0.23, *p* = .631, or in terms of parents' country of origin, *χ* ^2^s ≤ 9.39, *p*s ≥ .208, age, *t*(107) = −0.01, *p* = .996, or educational level, *t*(111) = 0.21, *p* = .837.

### 2.2 Procedure

#### 2.2.1 Social performance task

At the age of 4.5 years, children visited the lab with one of their parents, who was present throughout the procedure. A wooden stage was placed in the room, with a standing microphone and a spotlight in front. Children were told that they were going to sing a song on stage. They were asked to dress up with pop‐star‐like clothes and accessories that we provided, such as a shiny blouse and jacket. An unknown person entered the room while carrying a large, Hollywood‐style camera, and children were told that their performance would be videotaped.

Then, during the anticipation phase, children sat on the podium for 2 min. Next, during the performance phase, children stood on stage and sang a song of their own choosing in the presence of three audience members: their parent, the experimenter, and the camera person who videotaped the performance. Children were then introduced to the audience by the experimenter: “Let me introduce you to the audience. Ladies and gentlemen, today we have a special performance by the famous [child's first name], who will sing [name of song]!” Because children used a standing microphone, they did not move excessively (e.g., they did not dance or walk). The experimenter gently encouraged children to continue singing if they sang for less than 60 s. Children sang for an average of 82 s, *SD* = 45 (range = 17–270). Finally, during the recovery phase, children sat on the podium for 1 min. During the anticipation and recovery phase, children were not videotaped by the camera person.

#### 2.2.2 Physiological assessment

During the 2‐min anticipation, 1‐min performance, and 1‐min recovery periods, children's physiological responses were recorded and analyzed with Vsrrp98 software (Molenkamp, 2011). Data acquisition was performed by a National Instruments NI6224 data acquisition card, which sampled at a rate of 200S/s per channel. Electrocardiography was recorded using a standard Lead‐II configuration. R waves were automatically detected and corrected for artifacts. The raw ECG signal was filtered at a high‐pass frequency of 0.5 Hz, second‐order Butterworth. Next, a second‐order bandpass filter at 17 Hz was applied to extract the r‐tops from the signal. After filtering, the QRS detector was applied between 5 and 150 milliseconds. Heart rate was calculated as the number of R waves per minute. Heart rate variability was calculated as the square root of the mean squared differences (RMSSD) of successive normal‐to‐normal (NN) intervals (Malik, 1996). Electrodermal activity was recorded in micro‐Siemens with two curved Ag/AgCl electrodes placed on the middle phalanx of the middle and index finger of the child's left hand. Invalid recordings (e.g., heart rate > 220) were coded as missing data points.

#### 2.2.3 Narcissism and self‐esteem

Three years after the lab visit, at the age of 7.5, children were invited to complete questionnaires. An experimenter provided children with instructions and was present to answer children's questions. Children completed the questionnaires individually, in silence. Narcissism was assessed using the 10‐item Childhood Narcissism Scale (CNS; Thomaes et al., 2008), which assesses narcissism as single, unified personality trait. Sample items include: “I think it's important to stand out” and “I am a very special person” (1 = *not at all true* to 4 = *completely true*). The CNS, a continuous measure, is the most frequently used scale to assess children's narcissism as a non‐clinical, everyday personality trait (rather than as a disorder; Thomaes & Brummelman, 2016), and it has been validated extensively for use in Dutch children (e.g., Brummelman et al., 2018; Thomaes et al., 2008). Responses were averaged across items (Cronbach's α = .78), with higher scores indicating higher levels of narcissism. Average scores ranged from 1.00 to 4.00 (*M* = 2.30, *SD* = 0.67) and reflect adequate normality (skewness = 0.55, *SE* = 0.25; kurtosis = −0.05, *SE* = 0.50). The average level of narcissism in our sample is consistent with other recent studies on narcissism in community samples of children (Brummelman et al., 2018; Brummelman et al., 2021; Brummelman, Thomaes, Nelemans, Orobio de Castro, Overbeek, & Bushman, 2015; Grapsas et al., 2021). Rather than classifying children as narcissistic or non‐narcissistic, we analyzed narcissism as a continuum, ranging from low to high levels.

Self‐esteem was assessed using the six‐item Global Self‐Worth Subscale of the Self‐Perception Profile for Children (SPPC; Harter, 1985). Sample items include: “Some kids are happy with themselves as a person” and “Some kids like the kind of person they are.” Following others (e.g., Brendgen et al., 2004; Thomaes et al., 2008), we used a simplified response format with a 4‐point Likert scale (1 = *I am not like these kids at all* to 4 = *I am exactly like these kids*) rather than a more complex “some/other” response format (Yeager & Krosnick, 2012). The SPPC, a continuous measure, is the most frequently used scale to assess children's self‐esteem, and it has been validated extensively for use in Dutch children (e.g., Muris et al., 2003; Van Dongen‐Melman et al., 1993). Responses were averaged across items (Cronbach's α = .65), with higher scores indicating higher levels of self‐esteem. Average scores ranged from 1.50 to 4.00 (*M* = 3.29, *SD* = 0.55) and reflect slight non‐normality (skewness = −0.85, *SE* = 0.25; kurtosis = 0.59, *SE* = 0.50). This average level of self‐esteem in our sample was slightly lower than in other recent studies on self‐esteem in community samples of children (Grapsas et al., 2021; Harris et al., 2018).

### 2.3 Data analysis

Of the 113 children, two did not visit the lab at 4.5 years, 22 did not complete questionnaires at 7.5 years, and one completed the narcissism but not the self‐esteem questionnaire at 7.5 years. Of the 111 children who visited the lab at 4.5 years, eight refused to sing. Of those who sang, nine children had completely missing physiological data and seven children had missing physiological data for one or two task phases, both due to malfunctioning electrodes. There were no significant differences in narcissism or self‐esteem between children who did versus did not refuse to perform, *t*(87) = −1.01, *p* = .315 and *t*(12.13) = −0.16, *p* = .873, respectively. Also, there were no significant differences in narcissism, self‐esteem, or duration of performance between children whose electrodes did versus did not malfunction, *t*(80) = 0.06, *p* = .953, *t*(79) = −0.28, *p* = .779, and *t*(100) = −1.62, *p* = .109, respectively, or between children who did or did not have missing physiological data for some of the task phases, *t*(73) = −0.98, *p* = .330, *t*(72) = −0.57, *p* = .573, and *t*(91) = −0.67, *p* = .503, respectively.

To account for the nested structure of our data, we conducted our primary analyses using multilevel regression models with restricted maximum likelihood (REML) estimation including a random intercept, with task phase (Level 1: anticipation, performance, recovery) nested within individuals (Level 2), in IBM SPSS Statistics for Windows, Version 25.0. We did not include a random slope, since the variance of the slope was not significant and including it reduced model fit. Since these analyses account for missing data on the dependent variable, they included those children who had both (a) complete narcissism and self‐esteem data and (b) physiological data on at least one of the task phases, leading to a total of 71 children in each main analysis.

Because narcissism and self‐esteem were positively related, *r* = .33, *p* = .001, we included them in the same models, thus accounting for their shared variance. This is important because narcissism and self‐esteem consistently operate as mutual suppressors; in those cases, their unique associations with outcomes are revealed only when controlling for the other variable (Lawson & Robins, 2021; Paulhus et al., 2004). In our case, the analyses with narcissism as the main predictor included self‐esteem as a covariate, and vice versa. Narcissism and self‐esteem were standardized. We ran separate models for each dependent variable (i.e., heart rate, heart rate variability, and skin conductance). We first ran models to test the main effect of task phase, narcissism, and self‐esteem. We then ran separate models to test the narcissism × task phase and self‐esteem × task phase interactions. Significance level was set at .05, two‐tailed.

Our analyses include two dummy variables for task phase. Task phase dummy 1 compared the anticipation with the performance phase. Task phase dummy 2 compared the recovery with the performance phase. By including these dummy variables in our analyses, we were able to examine whether the associations of narcissism and self‐esteem with physiological responses differed between task phases.

## 3 RESULTS

Table 1 displays descriptive statistics and correlations. Correlations between heart rate, heart rate variability, and skin conductance levels were weak, attesting to their independence. For each physiological measure, correlations between anticipation, performance, and recovery phases were strong, indicating high test–retest reliability. There were no significant sex differences in narcissism, *t*(89) = −1.26, *p* = .209, self‐esteem, *t*(88) = −0.62, *p* = .539, or any of the physiological measures, *t*s ≤1.95, *p*s ≥ .055. Our multilevel models are presented in Tables 2 and 3, showing main effects (Tables 2 and 3: Models 1, 3, and 5), narcissism × task phase interactions (Table 2: Models 2, 4, and 6), and self‐esteem × task phase interactions (Table 3: Models 2, 4, and 6).

**TABLE 1:** Means (M), standard deviations (SD), and correlations among main variables

Variable	*M*	*SD*	1	2	3	4	5	6	7	8	9	10	11
1. Duration^a^	81.83	44.75	–										
2. HR ‐ anticipation	113.16	24.12	−.03	–									
3. HRV ‐ anticipation	41.55	20.16	.06	−.04	–								
4. SC ‐ anticipation	14.70	7.37	.11	.16	−.15	–							
5. HR ‐ performance	116.96	22.68	.03	.91**	−.15	.15	–						
6. HRV ‐ performance	37.45	24.05	.04	.16	.69**	−.10	.01	–					
7. SC ‐ performance	18.20	8.39	.12	.14	−.18	.94**	.12	−.12	–				
8. HR ‐ recovery	112.40	24.60	.12	.90**	−.05	.12	.91**	.14	.08	–			
9. HRV ‐ recovery	43.05	22.64	.01	−.01	.86**	−.10	−.15	.67**	−.09	−.16	–		
10. SC ‐ recovery	17.36	8.09	.20	.13	−.11	.88**	.10	.08	.93**	.07	−.07	–	
11. Narcissism	2.30	0.67	.10	−.04	−.02	.11	−.09	.05	−.03	−.08	−.03	.07	–
12. Self‐esteem	3.29	0.55	−.03	−.09	.09	−.38**	−.05	.07	−.39**	−.08	.00	−.30*	.33**

*Note. N* = 68–90.

Abbreviations: HR, heart rate; HRV, heart rate variability; SC, skin conductance.

* *p* < .05

** *p* < .01.

^a^ Duration of performance in minutes.

**TABLE 2:** Associations between task phase and physiological variables as a function of narcissism, controlling for self‐esteem

	Heart rate		Heart rate variability		Skin conductance	
	Model 1	Model 2	Model 3	Model 4	Model 5	Model 6
Intercept	117.18 (3.05)***	117.19 (3.05)***	37.23 (2.79)***	37.21 (2.79)***	17.04 (0.81)***	17.08 (0.81)***
Task phase dummy 1	−2.79 (1.29)*	−2.81 (1.29)*	**6.04 (1.92)** **	**6.07 (1.93)** **	**−3.09 (0.40)** ***	**−3.18 (0.40)** ***
Task phase dummy 2	**−4.08 (1.28)** **	**−4.09 (1.29)** **	**7.04 (1.91)** ***	**7.08 (1.92)** ***	−0.57 (0.40)	−0.62 (0.40)
Narcissism	−1.82 (3.14)	−2.07 (3.23)	0.04 (2.71)	1.02 (2.93)	1.25 (0.81)	0.74 (0.84)
Self‐esteem	−1.43 (3.06)	−1.43 (3.06)	1.35 (2.64)	1.34 (2.64)	**−3.05 (0.81)** ***	**−3.05 (0.81)** ***
Interaction						
Task phase dummy 1 × Narcissism		0.61 (1.31)		−1.22 (1.96)		0.98 (0.39)*
Task phase dummy 2 × Narcissism		0.16 (1.30)		−1.74 (1.95)		0.56 (0.39)
Marginal *R* ^*2*^	.014	.014	.021	.022	.185	.188
Conditional *R* ^*2*^	.916	.915	.776	.774	.902	.905

*Note*: Values from the multilevel models can be interpreted as unstandardized regression coefficients with standard errors given in parentheses. Task phase dummy 1 compared the anticipation with the performance phase. Task phase dummy 2 compared the recovery with the performance phase. Models 1, 3, and 5 are identical to those in Table 3. Parameter estimates with *p* < .005, based on *p* value correction, are indicated in bold.

* *p* < .05

** *p* < .01

*** *p* < .001.

**TABLE 3:** Associations between task phase and physiological variables as a function of self‐esteem, controlling for narcissism

	Heart rate		Heart rate variability		Skin conductance	
	Model 1	Model 2	Model 3	Model 4	Model 5	Model 6
Intercept	117.18 (3.05)***	117.16 (3.05)***	37.23 (2.79)***	37.22 (2.79)***	17.04 (0.81)***	17.03 (0.81)***
Task phase dummy 1	−2.79 (1.29)*	−2.75 (1.29)*	**6.04 (1.92)** **	**6.04 (1.93)** **	**−3.09 (0.40)** ***	**−3.08 (0.40)** ***
Task phase dummy 2	**−4.08 (1.28)** **	**−4.05 (1.28)** **	**7.04 (1.91)** ***	**7.09 (1.93)** ***	−0.57 (0.40)	−0.57 (0.40)
Narcissism	−1.82 (3.14)	−1.82 (3.14)	0.04 (2.71)	0.04 (2.71)	1.25 (0.81)	1.25 (0.81)
Self‐esteem	−1.43 (3.06)	−0.56 (3.14)	1.35 (2.64)	1.72 (2.86)	**−3.05 (0.81)** ***	**−3.32 (0.84)** ***
Interaction						
Task phase dummy 1 × Self‐esteem		−1.53 (1.26)		0.03 (1.90)		0.33 (0.39)
Task phase dummy 2 × Self‐esteem		−1.09 (1.27)		−1.19 (1.92)		0.50 (0.40)
Marginal *R* ^*2*^	.014	.015	.021	.022	.185	.185
Conditional *R* ^*2*^	.916	.916	.776	.773	.902	.902

*Note*: Values from the multilevel models can be interpreted as unstandardized regression coefficients with standard errors given in parentheses. Task phase dummy 1 compared the anticipation with the performance phase. Task phase dummy 2 compared the recovery with the performance phase. Models 1, 3, and 5 are identical to those in Table 2. Parameter estimates with *p* < .005, based on *p* value correction, are indicated in bold.

* *p* < .05

** *p* < .01

*** *p* < .001.

### 3.1 Preliminary analyses

The social performance task successfully induced physiological arousal (see Table 1 for means and standard deviations across task phases). There was a significant main effect of task phase for heart rate, *F*(2, 132.45) = 5.33, *p* = .006, heart rate variability, *F*(2, 133.63) = 7.93, *p* = .001, and skin conductance, *F*(2, 133.68) = 33.38, *p* < .001. On average, from anticipation to performance, heart rate increased, *B* = 2.79, 95% CI [0.24, 5.33], *t*(132.56) = 2.17, *p* = .032, *r* = .19, skin conductance increased, *B* = 3.09, 95% CI [2.29, 3.88], *t*(133.77) = 7.70, *p* < .001, *r* = .55, and heart rate variability decreased, *B* = −6.04, 95% CI[−9.84, −2.34], *t*(133.90) = −3.14, *p* = .002, *r* = .26. On average, from performance to recovery, heart rate decreased, *B* = −4.08, 95% CI [−6.61, −1.55], *t*(132.20) = −3.19, *p* = .002, *r* = .33, heart rate variability increased, *B* = 7.04, 95% CI [3.25, 10.83], *t*(133.00) = 3.68, *p* < .001, *r* = .52, and skin conductance did not change significantly, *B* = −0.57, 95% CI [−1.37, 0.23], *t*(133.39) = −1.41, *p* = .161, *r* = .12.

### 3.2 Primary analyses

#### 3.2.1 Narcissism

**FIGURE 1:**
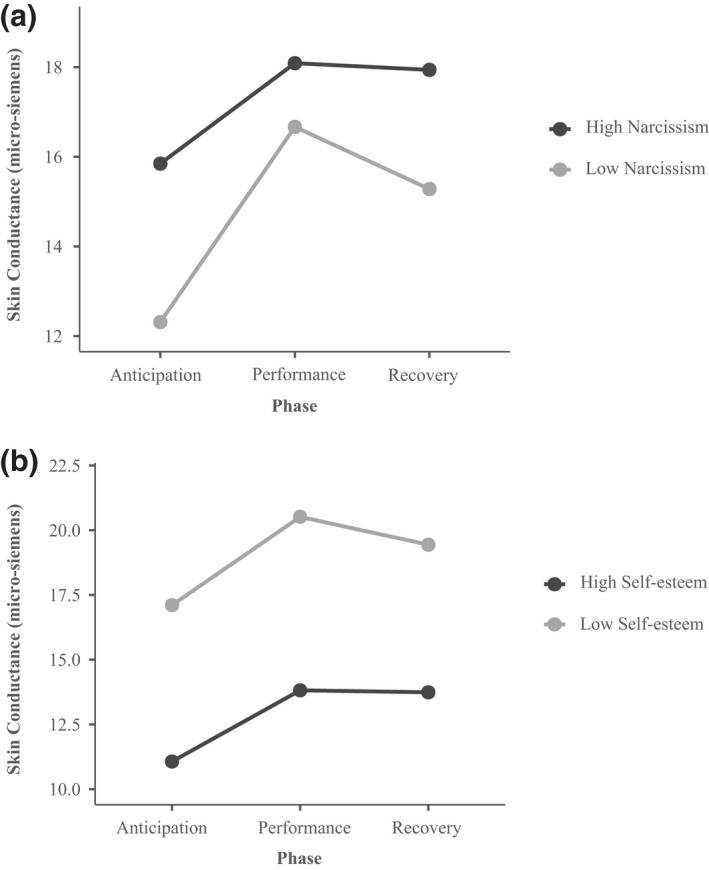
Panel a: The effect of task phase on skin conductance for children high in narcissism (1 *SD* above the mean) and low in narcissism (1 *SD* below the mean). Panel B: The effect of task phase on skin conductance for children with high self‐esteem (1 *SD* above the mean) and low self‐esteem (1 *SD* below the mean)

There were no significant main effects of narcissism on heart rate, heart rate variability, or skin conductance. The narcissism × task phase interaction was not significant for heart rate, *F*(2, 130.19) = 0.12, *p* = .889, or heart rate variability, *F*(2, 131.01) = 0.42, *p* = .656, but was significant for skin conductance, *F*(2, 131.33) = 3.26, *p* = .042 (Figure 1a). We conducted two follow‐up tests (Aiken & West, 1991).

First, we examined the association between narcissism and skin conductance within each task phase. Narcissism was associated with higher skin conductance during anticipation, *B* = 1.72, 95% CI [0.52, 3.39], *t*(78.56) = 2.05, *p* = .043, *r* = .23, but was not significantly related to skin conductance during performance, *B* = 0.73, 95% CI [−0.93, 2.40], *t*(78.36) = 0.88, *p* = .381, *r* = .10, and recovery, *B* = 1.29, 95% CI [−0.37, 2.96], *t*(78.37) = 1.55, *p* = .126, *r* = .17.

Second, we examined how skin conductance changed from anticipation to performance, and from performance to recovery, for children low (1 *SD* below the mean) and high (1 *SD* above the mean) in narcissism. Children with low narcissism levels showed a steep increase in skin conductance when going from anticipation to performance, *B* = 4.16, 95% CI [3.02, 5.31], *t*(131.65) = 7.19, *p* < .001, *r* = .53, and a modest decrease in skin conductance when going from performance to recovery, *B* = −1.18, 95% CI [−2.32, −0.03], *t*(131.37) = −2.03, *p* = .045, *r* = −.17. By contrast, children with high narcissism levels showed a smaller increase in skin conductance when going from anticipation to performance, *B* = 2.20, 95% CI [1.16, 3.24], *t*(131.45) = 4.18, *p* < .001, *r* = .34, and no significant change in skin conductance when going from performance to recovery, *B* = −0.06, 95% CI [−1.10, 0.98], *t*(131.30) = −0.12, *p* = .909, *r* = −.01.

Thus, in children predisposed to high narcissism levels, skin conductance was elevated during anticipation, rose significantly during performance (but less so than in other children), and remained elevated throughout recovery.

#### 3.2.2 Self‐esteem

There were no significant main effects of self‐esteem on heart rate or heart rate variability, but there was a significant main effect of self‐esteem levels on skin conductance, *F*(1, 68.02) = 14.09, *p* < .001, with self‐esteem levels being associated with lower skin conductance overall, *B* = −3.32, 95% CI [−5.00, −1.64], *t*(68.02) = −3.94, *p* < .001, *r* = −.43 (Figure 1b). There was no significant self‐esteem × task phase interaction for heart rate, *F*(2, 130.22) = 0.78, *p* = .461, heart rate variability *F*(2, 131.07) = 0.26, *p* = .771, or skin conductance, *F*(2, 131.45) = 0.79, *p* = .457 (Table 3). Thus, children predisposed to higher self‐esteem levels displayed lower skin conductance throughout the procedure.

The effect of self‐esteem was larger than the effect of narcissism. In terms of explained variance, the main effect of self‐esteem on skin conductance overall (17%) was more than twice as large as the effect of narcissism on skin conductance during anticipation (5%).

### 3.3 Robustness analyses

We examined the robustness of our findings in three ways.

First, because some children sang for less than 60 s, we repeated our analyses with 30‐s instead of 1‐min physiology assessments during performance (for details, see Supporting Information). These analyses show the same effects.

Second, because we conducted multiple tests per hypothesis, we divided the alpha level by the number of physiological measures times the number of task phases (three measures times three task phases), which resulted in a corrected alpha of .05/9 = .005 for our primary analyses. These analyses show that self‐esteem was still significantly related to lower skin conductance levels overall (results denoted in bold in Table 2), while the association between narcissism and skin conductance became statistically non‐significant.

Third, we repeated our main analyses for narcissism without controlling for self‐esteem, and our main analyses for self‐esteem without controlling for narcissism (for details, see Supporting Information). This did not change our results (i.e., no significant effect became non‐significant, and no nonsignificant effect became significant), with one exception: The association between narcissism and skin conductance within the anticipation phase became non‐significant, *B* = 0.75, 95% CI [−0.96, 2.45], *t*(80.10) = 0.87, *p* = .385, *r* = .10, showing that self‐esteem acts as a suppressor (Paulhus et al., 2004). Importantly, the narcissism × task phase interaction remained significant for skin conductance, *F*(2, 131.20) = 3.15, *p* = .046. As in the original analyses, children with high narcissism levels showed a blunted increase in skin conductance when going from anticipation to performance, and they showed no significant change in skin conductance when going from performance to recovery.

Together, these robustness analyses show that our results were robust across different specifications of performance but were generally more robust for self‐esteem than for narcissism.

## 4 DISCUSSION

Our prospective study is the first to examine the early physiological indicators of self‐esteem and narcissism. We built on social‐cognitive developmental theories of self‐esteem and narcissism (e.g., Brummelman & Sedikides, 2020; Tracy et al., 2009), which hold that self‐esteem and narcissism are rooted in distinct sets of socially relevant mental representations (e.g., beliefs, emotions, and action tendencies), which arise early in development and manifest physiologically in social‐evaluative contexts. We theorized that, in social‐evaluative contexts, children predisposed to high narcissism levels would show *elevated* physiological arousal, whereas children predisposed to high self‐esteem levels would show *lowered* physiological arousal. To test these hypotheses, we assessed children's physiological arousal during a social performance task at age 4.5 and assessed their self‐esteem and narcissism levels at age 7.5, when stable individual differences in self‐esteem and narcissism have emerged. Consistent with our theoretical predictions, children predisposed to higher narcissism levels showed elevated skin conductance levels during anticipation of the task; these levels remained elevated during performance and failed to return to baseline during recovery. By contrast, children predisposed to higher self‐esteem levels showed lowered skin conductance throughout the procedure. The effects were larger and more robust for self‐esteem than for narcissism, and they were specific to skin conductance; self‐esteem and narcissism were not significantly related to heart rate and heart rate variability. Together, these findings are consistent with the view that children predisposed to high narcissism levels are more fragile and prone to social‐evaluative concerns, whereas children predisposed to high self‐esteem levels are more secure and able to feel comfortable in social‐evaluative contexts.

### 4.1 Understanding narcissism versus self‐esteem

Over the past decades, the field has made significant progress in understanding the nature of narcissism and self‐esteem. Narcissism and self‐esteem both involve positive perceptions of the self, which explains why they were modestly related in the current study. Yet, they differ markedly in their phenotype, consequences, development, and origins (Brummelman et al., 2016; Campbell et al., 2002; Donnellan et al., 2005; Hyatt et al., 2018; Tracy et al., 2009). Extending this past work, our research shows that narcissism and self‐esteem have distinct early physiological indicators. While children predisposed to high narcissism levels showed elevated skin conductance while anticipating their on‐stage performance, children predisposed to high self‐esteem levels showed lowered skin conductance, not just during anticipation but throughout the procedure. Consistent with earlier work (e.g., Dieleman et al., 2015; Fabes et al., 1993), skin conductance was only weakly related to heart rate and heart rate variability. While skin conductance reflects sympathetic activation and is thought to underlie fight‐or‐flight responses (Kreibig, 2010), heart rate variability reflects parasympathetic withdrawal and is thought to underlie emotion regulation (Appelhans & Luecken, 2006). Heart rate reflects a mix of sympathetic activation and parasympathetic withdrawal (Kreibig, 2010). Together, these findings suggest that children predisposed to high narcissism levels may be more prone to fight‐or‐flight responses in social‐evaluative contexts, but they do not suggest that these children are less able to regulate their emotional responses. Children predisposed to high self‐esteem levels, on the other hand, do not show such fight‐or‐flight responses in social‐evaluative contexts.

Our results provide tentative evidence that narcissism, unlike self‐esteem, is reflected in early emerging physiological hyperarousal. This hyperarousal arose specifically during the anticipation of social exposure: While anticipating their on‐stage performance, children predisposed to high narcissism levels showed elevated skin conductance. During such a phase of anticipation, children predisposed to high narcissism levels may worry about how their upcoming performance will be evaluated by others, as narcissism is known to be related to a fear of negative evaluation (Thomaes et al., 2008) and to insecure attachment (Menon et al., 2018). Children predisposed to high narcissism levels did not, however, show elevated heart rate or heart rate variability. Unlike heart rate and heart rate variability, skin conduction is driven primarily by the sympathetic nervous system, which is involved in fight‐or‐flight responses (Kreibig, 2010). This suggests that children predisposed to high narcissism levels enter a fight‐or‐flight mode when they anticipate being in the center of attention. When maintained over long periods of time, such a response might have detrimental health consequences and help to explain why adults with high narcissism levels tend to have elevated basal oxidative stress levels (e.g., 8‐OH‐DG levels; Lee et al., 2020). It is important to emphasize that these effects of narcissism were small and not robust to correction for multiple testing, which emphasizes the need for highly powered replications.

Unlike children predisposed to high narcissism levels, those predisposed to high self‐esteem levels had reduced overall skin conductance levels. They started off with low levels of skin conductance, and these levels remained lower than those of children predisposed to low self‐esteem levels. To be sure, this does not mean that children predisposed to high self‐esteem levels were insensitive to social evaluation. In fact, their skin conductance levels rose from anticipation to performance, just as much as it did for children predisposed to low self‐esteem levels. Thus, these children seem to have a normative sensitivity to social evaluation. Together, these findings suggest that children predisposed to high self‐esteem levels were less stressed overall, perhaps because they did not expect others to evaluate them negatively. This interpretation concurs with the sociometer model of self‐esteem (Leary & Baumeister, 2000), which holds that self‐esteem serves as a gauge—or sociometer—that indexes one's perceived likelihood of being accepted and valued by others (Thomaes et al., 2010). Children with high self‐esteem levels believe that they will generally be accepted and valued (Leary & Baumeister, 2000), perhaps because they have internalized their parents' unconditional regard for them (Kernis et al., 2000) and they feel securely attached (Menon et al., 2018). Thus, even when they are preparing for or doing something as stressful as singing a song on stage in front of an audience while being videotaped, they may find comfort in the idea that others will most likely evaluate them favorably, giving rise to low skin conductance levels. Given that the effects of self‐esteem on skin conductance were substantial in size, were robust to different model specifications, and survived correction for multiple testing, our results seem to have uncovered a robust early physiological indictor of self‐esteem.

On average, children experienced a steep increase in skin conductance when going from anticipation to performance. Yet, this increase was attenuated for children predisposed to high narcissism levels. Why? One explanation is that these children were already high in skin conductance during anticipation, leaving less room for further increases (i.e., ceiling effect). Another explanation is that these children, despite fearing social evaluation, experience the performance phase as unambiguously positive, making it less threatening. Indeed, children were introduced in a grandiose way: “Ladies and gentlemen, today we have a special performance by the famous [child's first name], who will sing [name of song]!” Children with high narcissism levels may be used to being praised in inflated ways (Brummelman et al., 2017), and they enjoy being at the center of attention, but only when they experience the attention as unambiguously positive (Brummelman et al., 2018; Thomaes et al., 2010). In fact, it is possible that children predisposed to high narcissism levels experienced elevated skin conductance levels during positive social exposure as pleasant and sought to maintain those levels, which would explain why their skin conductance levels did not drop during recovery.

More broadly, our research adds to the idea that narcissism and self‐esteem are “sibling constructs.” Sibling constructs are empirically related, but are not identical; that is, they are not “twin constructs” (Lawson & Robins, 2021). Previous research has shown that narcissism and self‐esteem consistently function as mutual suppressor variables (Paulhus et al., 2004). A suppressor variable removes criterion‐irrelevant variance from the predictor (Horst, 1941), so that statistically controlling for a suppressor variable reveals a stronger association between the predictor and the criterion. Thus, when narcissism and self‐esteem are positively correlated (like in our study), it is important to control for self‐esteem to reveal the effects of narcissism, and vice versa (like we did). Self‐esteem was related to reduced skin conductance, regardless of whether we controlled for narcissism. However, narcissism was related to elevated skin conductance during anticipation, but only when we controlled for self‐esteem, showing that self‐esteem acted as a suppressor. In the case of suppression, statistical control can make self‐esteem and narcissism conceptually closer to the theorized constructs: “self‐esteem with narcissistic self‐aggrandizement removed is closer to genuine self‐esteem, whereas narcissism with self‐esteem removed is more like pure self‐aggrandizement, not just self‐confidence” (Lawson & Robins, 2021, p. 353). Without such an a priori theoretical justification, however, interpreting partial coefficients can be problematic, especially when constructs are highly correlated. In those cases, it is often difficult to know what a construct represents once variance shared with another construct is removed (Lynam et al., 2006; Sleep et al., 2017).

### 4.2 Uncovering the origins of social‐evaluative concerns

Consistent with social‐cognitive developmental theories of narcissism and self‐esteem (e.g., Brummelman & Sedikides, 2020; Tracy et al., 2009), our findings show that narcissism and self‐esteem may be underpinned by distinct ways of responding to social‐evaluative contexts. What might be the developmental origins of these ways of responding? Narcissism and self‐esteem are partly heritable (Neiss et al., 2002; Vernon et al., 2008), so it is possible that their underpinnings are transmitted genetically. At the same time, there is growing evidence that narcissism and self‐esteem are also shaped through socialization (for overviews, see Brummelman & Sedikides, 2020; Thomaes & Brummelman, 2016). Narcissism can be cultivated, in part, by *parental overvaluation*—parent seeing their child as more special and more entitled than others (Brummelman, Thomaes, Nelemans, Orobio de Castro, Overbeek, & Bushman, 2015; Derry, 2018). Overvaluing parents generally do not approve of their children unconditionally; rather, they tend to make their regard conditional on children living up to their narcissistic standards (Brummelman & Sedikides, 2020). For example, these parents indicate, “I would find it disappointing if my child was just a ‘regular’ child” (Brummelman, Thomaes, Nelemans, Orobio de Castro, & Bushman, 2015, p. 678). Based on such experiences, children may become concerned that they may fail to live up to others' expectations of them, leading to social‐evaluative concerns. By contrast, self‐esteem can be cultivated, in part, by *parental warmth*—parents spending time with their children, showing interest in their activities, and sharing joy with them (Brummelman, Thomaes, Nelemans, Orobio de Castro, Overbeek, & Bushman, 2015; Harris et al., 2017). Warm parents tend to approve of their children unconditionally, for better and worse (Assor et al., 2004; Brummelman, 2018). Based on such experiences, children may learn that they are valuable for who they are, lowering their social‐evaluative concerns. Future research should disentangle the genetic and environmental contributions to narcissism, self‐esteem, and their underpinnings.

More broadly, the current research suggests that physiological responses to socially meaningful contexts in early childhood can foreshadow individual differences in personality development. According to cognitive‐affective processing systems theory (Mischel & Shoda, 1995, 2008), variation of individuals' behavior across situations arises from stable and distinctive *if*… *then*… contingencies. For example, children predisposed to high narcissism levels may not experience social‐evaluation concerns invariably; rather, they may experience such concerns specifically during the anticipation of social exposure (“*If* people evaluate me, *then* I fear their disapproval”; also see Morf & Horvath, 2010). By contrast, children predisposed to high self‐esteem levels may lack these contingencies, which would help explain why they tend to experience few social‐evaluative concerns (Baldwin & Sinclair, 1996; Kernis et al., 2000). An exciting research direction will be to use precise physiological assessments of *if*… *then*… contingencies in early childhood to examine whether and how these contingencies serve as building blocks of long‐term personality development. Especially when children are too young to provide reliable self‐reports of emotional states or behavioral inclinations, physiological assessments may provide a unique window into *if*… *then*… contingencies (Grapsas et al., 2021). Such physiological assessments can be complemented with other methods that circumvent narcissistic impression management strategies (Paulhus & Vazire, 2007), such as neuroimaging (Cascio et al., 2015; Jauk et al., 2017) and neuro‐endocrine assessment (Edelstein et al., 2010; Reinhard et al., 2012).

### 4.3 Strengths, limitations, and research directions

Our study has several methodological strengths, including its prospective design, its multi‐method assessment of physiological arousal, and its precise developmental timing. Our study also has limitations. First, our sample was modest in size. Our total sample size would have provided sufficient statistical power (.80 at α = .05, two‐tailed). However, due to the challenges of conducting intensive research with young children (e.g., children refusing to sing a song on stage), there was some data loss, and our final sample size fell short of our intended sample size. For this reason, we conducted robustness analyses and interpreted our findings cautiously. We call for well‐powered and preregistered replications of our findings.

Second, building on meta‐analytic evidence (Seddon et al., 2020), we theorized that the performance task would elicit social‐evaluative concerns, leading to physiological fight‐or‐flight responses. Consistent with this notion, physiological arousal increased substantially from anticipation to performance. However, the task might not have been distressing to all children (e.g., some children might have experienced the performance phase as a *challenge* rather than a *threat*; Coleman et al., 2019; Seery, 2013). To understand whether increased physiological arousal maps onto fight‐or‐flight responses, future research should examine children in settings that are unambiguously threatening (e.g., singing a song on stage while the audience is frowning and refraining from positive feedback). We call for research to address these questions.

Third, there was a 3‐year interval between our study's first and second wave. This was intentional, as it enabled us to examine, prospectively, which physiological indicators would predict the developmental emergence of narcissism and self‐esteem. However, we did not study the developmental mechanisms through which physiological indicators (observed at age 4.5) can develop into individual differences in narcissism and self‐esteem (observed at age 7.5). We call for research that unravels these developmental mechanisms (e.g., using intensive longitudinal designs, with monthly, weekly, or even daily assessments of presumed developmental mechanisms).

Fourth, during the anticipation phase, children already knew that they would be invited to sing a song on stage. This enabled us to investigate children's physiological arousal during the anticipation of social exposure. However, we did not have a neutral baseline phase during which children neither experienced nor anticipated social exposure. We call for future research that includes a neutral baseline phase, so as to establish whether the elevated skin conductance levels of children with high narcissism levels are unique to the anticipation of social exposure or reflect these chronically elevated levels of arousal.

Our findings also generate new research questions. We call for research on the early manifestations of narcissism and self‐esteem. We assessed narcissism and self‐esteem at the critical age of 7.5, when individual differences in both narcissism and self‐esteem can be assessed reliably (Thomaes & Brummelman, 2016). Despite the growing evidence that even younger children can evaluate their global worth as a person (Cimpian, 2017; Cimpian et al., 2017), there are no available measures of narcissism in younger children (Harris et al., 2018). We call for research to develop such measures, and to uncover the social‐cognitive processes that underlie the early development of narcissism and self‐esteem.

## 5 CONCLUSION

Promoting children's self‐esteem is widely seen as an important societal goal (Brummelman, 2022; Orth & Robins, 2014), but experts have voiced concern that promoting self‐esteem can lead to narcissism. In the current work, we provide evidence for the notion that narcissism and self‐esteem are fundamentally distinct. Our research suggests that narcissism and self‐esteem have unique early physiological indicators, with narcissism being predicted by physiological hyperarousal during anticipation of social exposure and self‐esteem being predicted by an overall state of reduced arousal. The effects were more robust for self‐esteem than narcissism. Together, these findings corroborate theoretical models that separate narcissism from self‐esteem, and they suggest that children predisposed to high narcissism levels are prone to social‐evaluative concerns, whereas those predisposed to high self‐esteem levels tend to feel comfortable in social‐evaluative contexts. An important challenge for future work is to design interventions that target these developmental mechanisms to promote self‐esteem without breeding narcissism.

## AUTHOR CONTRIBUTIONS

**Eddie Brummelman:** Conceptualization; methodology; writing‐original draft; writing‐review & editing. **Milica Nikolic:** Conceptualization; data curation; investigation; methodology; software; supervision; validation; writing – review and editing. **Barbara Nevicka:** Formal analysis; software; validation; visualization; writing – review and editing. **Susan M. Bögels:** Conceptualization; funding acquisition; methodology; project administration; resources; software; supervision; writing – review and editing.

## Supporting information


Supinfo

## Footnotes

^1^
These demographic data refer to 74 children. Each of our main analyses was conducted with a total of 71 children. Out of these 71 children, 68 were identical across analyses (i.e., these children had complete narcissism and self‐esteem data, as well as data on at least one of the task phases for all physiological measures), but the remaining children differed across analyses. Three children were included in the heart rate and heart rate variability analyses, because they had data on heart rate and heart rate variability (but not skin conductance). Three children were included in the skin conductance analyses, because they had data on skin conductance (but not heart rate and heart rate variability).
