# Supplementary material for: Early physiological indicators of narcissism and self‐esteem in children
Source: Psychophysiology. 2022 May 3;59(10):e14082. doi: 10.1111/psyp.14082 (PMC9542209; doi:10.1111/psyp.14082)
Supplement: Supplementary file 1 — Supinfo [file PSYP-59-e14082-s001.docx]

**Supporting Information**

**Robustness Analyses: Main Analyses With 30-Second Physiology Assessments**

Because some children sang for less than 60 seconds, we repeated our analyses with 30-second instead of 1-minute physiology assessments during performance. The results remained the same for all the analyses, attesting to their robustness.

Table S1 displays descriptive statistics and correlations. Correlations between heart rate, heart rate variability, and skin conductance levels were weak, attesting to their independence. For each physiological variable, correlations between anticipation, performance, and recovery phases were strong, indicating strong test-retest reliability. There were no significant gender differences in narcissism, self-esteem, or any of the physiological variables. Our multilevel models are presented in Table S2 and S3, showing main effects (Tables S2 and S3: Models 1, 3, and 5), narcissism × task phase interactions (Table S2: Models 2, 4, and 6), and self-esteem × task phase interactions (Table S3: Models 2, 4, and 6).

**Preliminary Analyses**

The social performance successfully induced physiological arousal (see Table S1 for means and standard deviations across task phases). There was a significant main effect of task phase for heart rate, *F*(2, 130.06) = 4.16, *p* = .018, heart rate variability, *F*(2, 132.75) = 7.28, *p* = .001, and skin conductance, *F*(2, 133.71) = 34.60, *p* < .001. On average, heart rate and skin conductance increased from anticipation to performance, *B* = 2.60, 95% CI [-0.15, 5.35], *t*(130.05) = 1.87, *p* = .063, *r* = .16, and *B* = 3.30, 95% CI [2.48, 4.11], *t*(133.80) = 7.97, *p* < .001, *r* = .57, respectively, whereas heart rate variability decreased from anticipation to performance, *B* = -6.31, 95% CI[-10.47, -2.16], *t*(132.27) = 3.00, *p* = .003, *r* = .25. Moreover, heart rate and skin conductance decreased from performance to recovery, *B* = -3.94, 95% CI [-6.69, -1.19], *t*(129.87) = -2.84, *p* = .005, *r* = .33, and *B* = -0.77, 95% CI [-1.59, 0.05], *t*(133.41) = -1.85, *p* = .066, *r* = .16, respectively, and heart rate variability increased from performance to recovery, *B* = 7.43, 95% CI [3.27, 11.59], *t*(132.27) = 3.53, *p* = .001, *r* = .29. Thus, the social performance task was effective at inducing physiological arousal.

**Primary Analyses**

**Narcissism.** There were no significant main effects of narcissism on heart rate, heart rate variability, or skin conductance. The narcissism × task phase interaction was not significant for heart rate, *F*(2, 127.70) = 0.24, *p* = .785, or heart rate variability, *F*(2, 129.90) = 0.83, *p* = .439, but it was significant for skin conductance, *F*(2, 131.34) = 3.41, *p* = .036. We conducted two follow-up tests (Aiken & West, 1991).

First, we examined the association between narcissism and skin conductance within each task phase. Narcissism was associated with higher skin conductance during anticipation, *B* = 1.73, 95% CI [0.04, 3.41], *t*(78.95) = 2.04, *p* = .045, *r* = .22, but was not significantly related to skin conductance during performance, *B* = 0.70, 95% CI [-0.99, 2.38], *t*(78.74) = 0.82, *p* = .415, *r* = .09, and recovery, *B* = 1.30, 95% CI [-0.39, 2.99], *t*(78.76) = 1.53, *p* = .129, *r* = .17.

Second, we examined how skin conductance changed from anticipation to performance, and from performance to recovery, for children low (1 SD below the mean) and high (1 SD above the mean) in narcissism. Children with low narcissism levels showed a steep increase in skin conductance when going from anticipation to performance, *B* = 4.43, 95% CI [3.25, 5.61], *t*(131.67) = 7.43, *p* < .001, *r* = .54, and a modest decrease in skin conductance when going from performance to recovery, *B* = -1.43, 95% CI [-2.61, -0.25], *t*(131.39) = -2.40, *p* = .018, *r* = .20. By contrast, children with high narcissism levels showed a smaller increase in skin conductance when going from anticipation to performance, *B* = 2.36, 95% CI [1.44, 4.72], *t*(131.46) = 4.35, *p* < .001, *r* = .35, and no significant decrease in skin conductance when going from performance to recovery, *B* = -0.22, *t*(131.31) = -0.40, 95% CI [-1.29, 0.85], *p* = .687, *r* = .03.

Thus, in children with high narcissism levels, skin conductance was elevated during anticipation, rose significantly during performance (but less so than in other children), and remained elevated throughout recovery.

**Self-esteem.** There were no main effects of self-esteem on heart rate or heart rate variability, but there was a significant main effect of self-esteem levels on skin conductance, *F*(1, 68.03) = 13.99, *p* < .001, with self-esteem levels being associated with lower skin conductance overall, *B* = -3.40, 95% CI [-5.10, -1.70], *t*(68.03) = -3.98, *p* < .001, *r* = .43. There was no self-esteem × task phase interaction for heart rate, *F*(2, 127.71) = 0.55, *p* = .580, heart rate variability *F*(2, 129.93) = 0.23, *p* = .795, or skin conductance, *F*(2, 131.47) = 1.02, *p* = .365 (Table S3). Thus, children with higher self-esteem displayed lower skin conductance throughout the procedure.

**Robustness Analyses: Main Analyses Without Covariates**

In line with robustness analyses, we repeated our main analyses for narcissism without controlling for self-esteem, and our main analyses for self-esteem without controlling for narcissism.

**Primary Analyses**

**Narcissism.** There were no significant main effects of narcissism on heart rate, heart rate variability, or skin conductance. The narcissism × task phase interaction was not significant for heart rate, *F*(2, 132.16) = 0.11, *p* = .897, or heart rate variability, *F*(2, 133.00) = 0.46, *p* = .636, but was significant for skin conductance, *F*(2, 131.20) = 3.15, *p* = .046. We conducted two follow-up tests (Aiken & West, 1991).

First, we examined the association between narcissism and skin conductance within each task phase. Narcissism was not significantly associated with skin conductance during anticipation, *B* = 0.75, 95% CI [-0.96, 2.45], *t*(80.10) = 0.87, *p* = .385, *r* = .10, performance, *B* = -0.21, 95% CI [-1.92, 1.49], *t*(79.93) = -0.25, *p* = .804, *r* = .03, or recovery, *B* = 0.35, 95% CI [-1.36, 2.05], *t*(80.03) = 0.40, *p* = .687, *r* = .04.

Second, we examined how skin conductance changed from anticipation to performance, and from performance to recovery, for children low (1 *SD* below the mean) and high (1 *SD* above the mean) in narcissism. Children with low narcissism levels showed a steep increase in skin conductance when going from anticipation to performance, *B* = 4.18, 95% CI [3.04, 5.32], *t*(133.47) = 7.25, *p* < .001, *r* = .53, and a modest decrease in skin conductance when going from performance to recovery, *B* = -1.18, 95% CI [-2.32, -0.04], *t*(133.24) = -2.04, *p* = .043, *r* = -.17. By contrast, children with high narcissism levels showed a smaller increase in skin conductance when going from anticipation to performance, *B* = 2.25, 95% CI [1.23, 3.29], *t*(133.30) = 4.34, *p* < .001, *r* = .35, and no significant change in skin conductance when going from performance to recovery, *B* = -0.06, 95% CI [-1.09, 0.97], *t*(133.17) = -0.12, *p* = .905, *r* = -.01.

Thus, in children predisposed to high narcissism levels, skin conductance rose significantly during performance (but less so than in other children), and remained elevated throughout recovery (unlike that of other children).

**Self-esteem.** There were no significant main effects of self-esteem on heart rate or heart rate variability, but there was a significant main effect of self-esteem levels on skin conductance, *F*(1, 69.01) = 11.64, *p* = .001, with self-esteem levels being associated with lower skin conductance overall, *B* = -2.65, 95% CI [-4.21, -1.10], *t*(69.01) = -3.41, *p* = .001, *r* = -.38. There was no significant self-esteem × task phase interaction for heart rate, *F*(2, 130.18) = 0.78, *p* = .461, heart rate variability *F*(2, 131.06) = 0.26, *p* = .771, or skin conductance, *F*(2, 131.44) = 0.79, *p* = .457 (Table 3). Thus, children predisposed to higher self-esteem levels displayed lower skin conductance throughout the procedure.

Table S1

*Means (M), Standard Deviations (SD), and Correlations Among Main Variables*

| Variable | *M* | *SD* | 1 | 2 | 3 | 4 | 5 | 6 | 7 | 8 | 9 | 10 | 11 |
| --- | --- | --- | --- | --- | --- | --- | --- | --- | --- | --- | --- | --- | --- |
| 1. Duration^a^ | 81.83 | 44.75 | — |  |  |  |  |  |  |  |  |  |  |
| 2. HR - anticipation | 113.16 | 24.12 | -.03 | — |  |  |  |  |  |  |  |  |  |
| 3. HRV - anticipation | 41.55 | 20.16 | .06 | -.04 | — |  |  |  |  |  |  |  |  |
| 4. SC - anticipation | 14.70 | 7.37 | .11 | .16 | -.15 | — |  |  |  |  |  |  |  |
| 5. HR - performance | 115.79 | 22.05 | -.02 | .90** | -.17 | .20 | — |  |  |  |  |  |  |
| 6. HRV - performance | 37.04 | 24.61 | .04 | .17 | .66** | -.08 | -.03 | — |  |  |  |  |  |
| 7. SC - performance | 18.39 | 8.56 | .11 | .14 | .18 | .94** | .16 | -.08 | — |  |  |  |  |
| 8. HR - recovery | 112.40 | 24.60 | .12 | .90** | -.05 | .12 | .85** | .16 | .07 | — |  |  |  |
| 9. HRV - recovery | 43.05 | 22.64 | .01 | -.01 | .86** | -.10 | -.09 | .62** | -.09 | -.16 | — |  |  |
| 10. SC - recovery | 17.36 | 8.09 | .20 | .13 | -.11 | .88** | .14 | -.05 | .93** | .07 | -.07 | — |  |
| 11. Narcissism | 2.30 | 0.67 | .10 | -.04 | -.02 | .11 | -.00 | .08 | -.03 | -.08 | -.03 | .07 | — |
| 12. Self-esteem | 3.29 | 0.55 | -.03 | -.09 | .09 | -.38** | -.04 | .07 | -.39** | -.08 | .00 | -.30* | .33** |

*Note.* *N* = 68-90; ^a^duration of performance in minutes; HR = heart rate, HRV = heart rate variability, SC = skin conductance.

**p* < .05. ***p* < .01.

Table S2

*Associations between Task Phase and Physiological Measures as a Function of Narcissism, Controlling for Self-Esteem*

|  | Heart Rate | |  | Heart Rate Variability | |  | Skin Conductance | |
| --- | --- | --- | --- | --- | --- | --- | --- | --- |
|  | Model 1 | Model 2 |  | Model 3 | Model 4 |  | Model 5 | Model 6 |
| Intercept | 117.01 (3.11)^***^ | 116.99 (3.11)^***^ |  | 36.87 (2.84)^***^ | 36.81 (2.84)^***^ |  | 17.25 (0.82)^***^ | 17.30 (0.82)^***^ |
| Task phase dummy 1 | –2.60 (1.39)^†^ | –2.58 (1.40)^†^ |  | **6.31 (2.10)^**^** | **6.38 (2.10)^**^** |  | **–3.30 (0.41)^***^** | **–3.40 (0.41)^***^** |
| Task phase dummy 2 | **–3.94 (1.39)^**^** | –3.91 (1.40)^**^ |  | **7.43 (2.10)^**^** | **7.51 (2.11)^**^** |  | –0.77 (0.42)^†^ | –0.83 (0.41)^*^ |
| Narcissism | –1.41 (3.18) | –0.89 (3.28) |  | 0.36 (2.70) | 1.90 (2.97) |  | 1.24 (0.82) | 0.70 (0.85) |
| Self-esteem | –1.57 (3.10) | –1.57 (3.10) |  | 1.25 (2.64) | 1.25 (2.64) |  | **–3.08 (0.82)^***^** | **–3.08 (0.82)^***^** |
| Interaction |  |  |  |  |  |  |  |  |
| Task phase dummy 1 × Narcissism |  | –0.55 (1.41) |  |  | –2.02 (2.13) |  |  | 1.03 (0.40)^*^ |
| Task phase dummy 2 × Narcissism |  | –0.98 (1.41) |  |  | –2.60 (2.12) |  |  | 0.61 (0.40) |
| Marginal *R^2^* | .012 | .012 |  | .022 | .024 |  | .186 | .189 |
| Conditional *R^2^* | .906 | .905 |  | .740 | .740 |  | .899 | .902 |

*Note.* Values from the multilevel models can be interpreted as unstandardized regression coefficients with standard errors given in parentheses. Task phase dummy 1 compared the anticipation with the performance phase. Task phase dummy 2 compared the recovery with the performance phase. Models 1, 3 and 5 are identical to those in Table S3. Parameter estimates with *p* < .005, based on p-value correction, are indicated in bold. ^†^ *p* < .067. * *p* < .05. ** *p* < .01. *** *p* < .001.

Table S3

*Associations between Task Phase and Physiological Measures as a Function of Self-Esteem, Controlling for Narcissism*

|  | Heart Rate | |  | Heart Rate Variability | |  | Skin Conductance | |
| --- | --- | --- | --- | --- | --- | --- | --- | --- |
|  | Model 1 | Model 2 |  | Model 3 | Model 4 |  | Model 5 | Model 6 |
| Intercept | 117.01 (3.11)^***^ | 116.99 (3.11)^**^ |  | 36.87 (2.84)^***^ | 36.86 (2.84)^**^ |  | 17.25 (0.82)^***^ | 17.24 (0.82)^**^ |
| Task phase dummy 1 | –2.60 (1.39)^†^ | –2.57 (1.39)^†^ |  | **6.31 (2.10)^**^** | **6.31 (2.11)^**^** |  | **–3.30 (0.41)^***^** | **–3.29 (0.41)^***^** |
| Task phase dummy 2 | **–3.94 (1.39)^**^** | –3.91 (1.40)^**^ |  | **7.43 (2.10)^**^** | **7.47 (2.12)^**^** |  | –0.77 (0.42)^†^ | –0.78 (0.42)^†^ |
| Narcissism | –1.41 (3.18) | –1.40 (3.18) |  | 0.36 (2.70) | 0.36 (2.70) |  | 1.24 (0.82) | 1.24 (0.82) |
| Self-esteem | –1.57 (3.10) | –0.79 (3.20) |  | 1.25 (2.64) | 1.60 (2.89) |  | **–3.08 (0.82)^***^** | **–3.40 (0.85)^***^** |
| Interaction |  |  |  |  |  |  |  |  |
| Task phase dummy 1 × Self-esteem |  | –1.40 (1.37) |  |  | 0.11 (2.07) |  |  | 0.41 (0.41) |
| Task phase dummy 2 × Self-esteem |  | –0.93 (1.38) |  |  | –1.18 (2.09) |  |  | 0.57 (0.42) |
| Marginal *R^2^* | .012 | .012 |  | .022 | .023 |  | .186 | .186 |
| Conditional *R^2^* | .906 | .905 |  | .740 | .737 |  | .899 | .899 |

*Note.* Values from the multilevel models can be interpreted as unstandardized regression coefficients with standard errors given in parentheses. Task phase dummy 1 compared the anticipation with the performance phase. Task phase dummy 2 compared the recovery with the performance phase. Models 1, 3 and 5 are identical to those in Table S2. Parameter estimates with *p* < .005, based on p-value correction, are indicated in bold. ^†^ *p* < .068. * *p* < .05. ** *p* < .01. *** *p* < .001.

Table S4

*Associations between Task Phase and Physiological Variables as a Function of Narcissism*

|  | Heart Rate | |  | Heart Rate Variability | |  | Skin Conductance | |
| --- | --- | --- | --- | --- | --- | --- | --- | --- |
|  | Model 1 | Model 2 |  | Model 3 | Model 4 |  | Model 5 | Model 6 |
| Intercept | 117.14 (2.99)^***^ | 117.15 (2.99)^***^ |  | 37.01 (2.75)^***^ | 36.98 (2.75)^***^ |  | 17.19 (0.86)^***^ | 17.24 (0.86)^***^ |
| Task phase dummy 1 | –2.83 (1.27)^*^ | –2.85 (1.28)^*^ |  | **6.00 (1.89)^**^** | **6.05 (1.90)^**^** |  | **–3.12 (0.40)^***^** | **–3.22 (0.39)^***^** |
| Task phase dummy 2 | **–4.10 (1.26)^**^** | **–4.10 (1.27)^**^** |  | **6.94 (1.89)^***^** | **7.00 (1.90)^***^** |  | –0.57 (0.40) | –0.62 (0.39) |
| Narcissism | –2.19 (3.00) | –2.43 (3.09) |  | 0.19 (2.61) | 1.20 (2.83) |  | 0.29 (0.83) | -0.21 (0.86) |
| Interaction |  |  |  |  |  |  |  |  |
| Task phase dummy 1 × Narcissism |  | 0.58 (1.30) |  |  | –1.24 (1.94) |  |  | 0.96 (0.38)^*^ |
| Task phase dummy 2 × Narcissism |  | 0.15 (1.29) |  |  | –1.80 (1.93) |  |  | 0.56 (0.38) |
| Marginal *R^2^* | .012 | .012 |  | .017 | .018 |  | .036 | .039 |
| Conditional *R^2^* | .915 | .914 |  | .775 | .774 |  | .901 | .904 |

*Note.* Values from the multilevel models can be interpreted as unstandardized regression coefficients with standard errors given in parentheses. Task phase dummy 1 compared the anticipation with the performance phase. Task phase dummy 2 compared the recovery with the performance phase. Parameter estimates with *p* < .005, based on p-value correction, are indicated in bold.

* *p* < .05. ** *p* < .01. *** *p* < .001.

Table S5

|  | Heart Rate | |  | Heart Rate Variability | |  | Skin Conductance | |
| --- | --- | --- | --- | --- | --- | --- | --- | --- |
|  | Model 1 | Model 2 |  | Model 3 | Model 4 |  | Model 5 | Model 6 |
| Intercept | 117.16 (3.04)^***^ | 117.14 (3.04)^**^ |  | 37.23 (2.77)^***^ | 37.22 (2.77)^**^ |  | 17.15 (0.81)^***^ | 17.14 (0.81)^**^ |
| Task phase dummy 1 | –2.79 (1.29)^*^ | –2.75 (1.29)^*^ |  | **6.04 (1.92)^**^** | **6.04 (1.93)^**^** |  | **–3.09 (0.40)^***^** | –3.08 (0.40)^**^ |
| Task phase dummy 2 | **–4.08 (1.28)^**^** | **–4.05 (1.28)^**^** |  | **7.04 (1.91)^***^** | **7.09 (1.93)^***^** |  | –0.57 (0.40) | –0.57 (0.40) |
| Self-esteem | –1.85 (2.96) | –0.98 (3.04) |  | 1.35 (2.55) | 1.73 (2.77) |  | **–2.65 (0.78)^**^** | **–2.92 (0.81)^**^** |
| Interaction |  |  |  |  |  |  |  |  |
| Task phase dummy 1 × Self-esteem |  | –1.53 (1.26) |  |  | 0.03 (1.90) |  |  | 0.33 (0.39) |
| Task phase dummy 2 × Self-esteem |  | –1.09 (1.28) |  |  | –1.19 (1.92) |  |  | 0.49 (0.40) |
| Marginal *R^2^* | .010 | .011 |  | .021 | .022 |  | .161 | .162 |
| Conditional *R^2^* | .915 | .915 |  | .773 | .771 |  | .901 | .901 |

*Associations between Task Phase and Physiological Variables as a Function of Self-Esteem*

*Note.* Values from the multilevel models can be interpreted as unstandardized regression coefficients with standard errors given in parentheses. Task phase dummy 1 compared the anticipation with the performance phase. Task phase dummy 2 compared the recovery with the performance phase. Parameter estimates with *p* < .005, based on p-value correction, are indicated in bold.

* *p* < .05. ** *p* < .01. *** *p* < .001.
